# Light quality as a driver in adapting photosynthetic acclimation to niche partitioning

**DOI:** 10.1093/jxb/erad409

**Published:** 2023-11-21

**Authors:** Eunchul Kim

**Affiliations:** Division of Environmental Photobiology, National Institute for Basic Biology, Myodaiji, Okazaki 444-8585, Japan; Department of Basic Biology, The Graduate University for Advanced Studies, SOKENDAI, Okazaki 444-8585, Japan

**Keywords:** Light quality, niche partitioning, *Ostreococcus*, photosynthetic acclimation

## Abstract

This article comments on:

**Sands E, Davies S, Puxty RJ, Verge V, Bouget F-Y, Scanlan DJ, Carre IA.** 2023. Genetic and physiological responses to light quality in a deep ocean ecotype of *Ostreococcus*, an ecologically important photosynthetic picoeukaryote. Journal of Experimental Botany **74**, 6773–6789.

***Ostreococcus*, one of the smallest green algae, is widely distributed in the ocean and plays a vital role as a primary producer in marine ecosystems. Its survival and growth are dependent on a myriad of factors, including light quality, which can vary as water movements transport them through different water depth and environments. Sands *et al.* (2023) studied the genetic and physiological responses to monochromatic light in two ecotypes of *Ostreococcus* habituated in shallow and deep oceans, respectively. This study offers insights into how genetic modifications can shape an organism’s ability to adapt to environmental niches, as well as the molecular mechanisms of photosynthetic acclimation in *Ostreococcus*.**

*Ostreococcus* belongs to the Prasinophyceae (Courties *et al.*, 1994, 1998) and is considered an ‘ancient’ green alga due to its proximity to the evolutionary junction between Chlorophyta and Streptophyta (Lewis and McCourt, 2004; Leliaert *et al.*, 2012) (Fig. 1A). Moreover, its simple structure―consisting of a single mitochondrion and chloroplast―and small genome size make it a suitable organism to explore the ‘bare limits’ of photosynthetic eukaryotes (Derelle *et al.*, 2006; Palenik *et al.*, 2007). Therefore, conducting a comprehensive study of the genetic and physiological traits of *Ostreococcus* and comparing them with those of other plant species can contribute to broadening our understanding of plant evolution and photosynthetic acclimations.

**Fig. 1.**
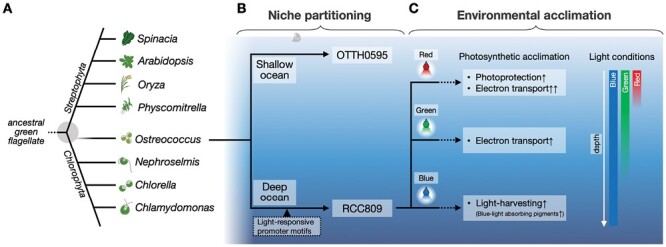
Hypothetical model for the different photosynthetic acclimation of two *Ostreococcus* ecotypes. (A) Schematic phylogenetic tree illustrating the representative genera of *Chlorophyta* and *Streptophyta* in the green lineages. (B) Hypothetical illustration of ecological niche partitioning of the two *Ostreococcus* ecotypes, OTTH0595 and RCC809, based on their respective origins and genomic differences in the light-responsive promoter motifs. (C) Photosynthetic acclimation of RCC809 in response to monochromatic light conditions. Conceptual illustration of light conditions (quality and quantity) as a function of depth in the ocean.

## Distinct responses to light quality in two *Ostreococcus* ecotypes

In Sands *et al.* (2023), two *Ostreococcus* ecotypes―the clade B species, RCC809, isolated from the deeper euphotic zones of the tropical Atlantic Ocean, and the clade A species, OTTH0595, isolated from shallow water habitats― showed different physiological responses during the long-term acclimation to monochromatic light conditions (Fig. 1B). The results in conjunction with the environmental conditions under which the two ecotypes are isolated suggest that the physiological responses in the two ecotypes are closely linked to their environmental niche partitioning.

RCC809 showed remarkable photosynthetic acclimation to the monochromatic light conditions (Fig. 1C). Under blue light treatment, it showed favorable acclimation to enhance light absorption by accumulating blue light-absorbing pigments, such as dihydrolutein, micromonal, Mg-2,4-divinylpheoporphyrin a_5_ (MgDVP), and Chl *b*. Simultaneously, the electron transport capacity was down-regulated, while cell division was up-regulated. This acclimation strategy is thought to optimize growth opportunities in environments with dim blue light conditions, such as the deep ocean. In contrast, red light conditions induced an increase in photoprotective capacity, specifically the non-photochemical quenching (NPQ), of RCC809. This acclimation is thought to mitigate the potential damage caused by excessive light when flowing into shallow ocean with high light intensity and the presence of red light. Green light conditions induced an intermediate state between blue and red light conditions.

OTTH0595 exhibited a more restrained response to the monochromatic light conditions compared with RCC809. This contrasting behavior between the two ecotypes has been proposed to be attributed to genetic differences originating from ecological niche partitioning, specifically the presence of certain light-responsive promoter motifs that are abundant in the RCC809 genome but not in OTTH0595.

## Perspective on photosynthetic acclimation: a closer look at light quality-dependent DEGs

In order to investigate the physiological responses to light quality, Sands *et al*. analyzed differentially expressed genes (DEGs) that depend on monochromatic light conditions. The results provide insight into the physiological processes including cell division cycle, photosynthetic carbon fixation, glycolysis and gluconeogenesis, chlorophyll and carotenoid biosynthesis, as well as accumulation of photoreceptors. These findings align with previous studies that have demonstrated photosynthetic adaptations, such as a linear correlation between NPQ and the deoxidation state of xanthophyll in *Ostreococcus* (Cardol *et al.*, 2008; Six *et al*., 2009). Despite these findings, the precise molecular mechanism governing photosynthetic acclimation—in particular, modulation of the maximum quantum yield of PSII and photoprotective NPQ—in *Ostreococcus*, remains unclear.

**Fig. 2.**
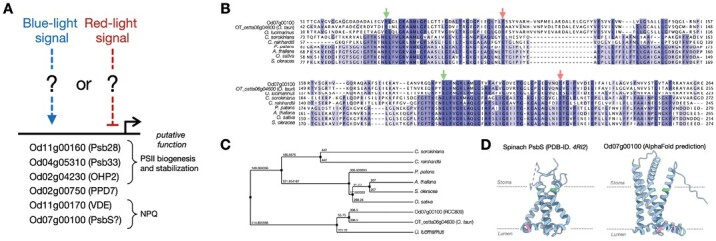
Light quality-dependent DEGs that potentially contribute to photosynthetic acclimation of PSII in *Ostreococcus*. (A) List of reported DEGs that may play a role in PSII biogenesis and stabilization, as well as NPQ. (B) Multiple sequence alignment (by using Clustal Omega) of putative PsbS from RCC809 (Od07g00100) and *O. tauri* (ostta06g04600) with PsbS from other species. Coloration is used to indicate the percentage identity. The green arrows denote two highly conserved glutamate residues that serve as binding sites for chlorophylls in LHCII, while the red arrows point to two glutamate residues necessary for qE quenching. (C) Phylogenetic tree illustrating average distances based on the polypeptide sequences of various PsbSs, including the putative PsbS. The analysis omits the extra-long C-terminal region of *C. sorokiniana* PsbS. (D) Structural comparison of spinach PsbS (PDB-ID. *4RI2*) and Od07g00100 (AlphaFold prediction model without transit peptides predicted by TargetP-2.0). The red and green segments correspond to the glutamate residues denoted by the red and green arrows in (B).

Sands *et al*. (2023) report six DEGs that may be associated with the photosynthetic acclimation of PSII. These genes were found to be up-regulated by blue light or down-regulated by red light (Fig. 2A). Among the six DEGs, four are regarded to be related to PSII core complexes. They include homologs of Psb28 (AT4G28660), Psb33 (AT1G71500), and OHP2 (AT1G34000), which are involved in the biogenesis and stability of PSII (Theis and Schroda, 2016; Hey and Grimm, 2018), as well as PPD7 (AT3G05410), which has an unknown function in *Arabidopsis thaliana*. A further DEG is the homolog of violaxanthin de-epoxidase (VDE) (AT1G08550) which is involved in NPQ by accumulating zeaxanthin (Niyogi *et al*., 1998). The remaining DEG Od07g00100 has highest homology with *A. thaliana* PsbS (AT1G44575), a pivotal protein for NPQ (Niyogi *et al.*, 2005), and is thus believed to be related to photoprotection. Despite a relatively low identity (32%) and an E-value of 1e-09, Od07g00100 conserves key functional domains of *A. thaliana* PsbS—two pH-sensing glutamate residues on the luminal side (indicated by red arrows in Fig. 2B) (Krishnan-Schmieden *et al.*, 2021) and two charge-compensated glutamates that serve as binding sites for chlorophylls in light-harvesting complex II (LHCII; indicated by green arrows in Fig. 2B) (Niyogi *et al.*, 2005). The phylogenetic tree of PsbS with putative PsbS shows that the putative PsbS is conserved in the *Ostreococcus* genus and forms a separate group from PsbS in other species (Fig. 2C). In terms of the protein structure, the predicted structural model of Od07g00100 (by AlphaFold) exhibits a rather different shape compared with spinach PsbS (Fig. 2D). It might be related to the different behavior of Od07g00100 compared with PsbS in other species. For example, Od07g00100 is up-regulated under blue light conditions, but with a decrease in the overall NPQ. Thus, investigating the functions of DEGs, particularly Od07g00100, is necessary to comprehend the process of photosynthetic acclimation in *Ostreococcus*.

## Stepping forward: elucidating photosynthetic acclimation in *Ostreococcus*

The investigation by Sands *et al.* (2023) suggests potential key players that modulate light-harvesting systems and PSII in *Ostreococcus*. It is noteworthy that the expression of genes involved in photoprotection, such as VDE and PsbS-like protein, was up-regulated under blue light conditions (or down-regulated under red light conditions). However, in contrast to other organisms, this led to a decrease in photoprotective capacity.

This contradictory result suggests two plausible scenarios: (i) the role of VDE and putative PsbS differs significantly from that documented in other organisms, or (ii) other factors, apart from VDE and PsbS, play a more significant role in photoprotection. Potential candidates that could be implicated in this process might include LHC stress-related proteins (LHCSRs)-dependent NPQ (Pinnola, 2019), state transitions (Minagawa, 2011), and configurational rearrangements of photosynthetic proteins on the thylakoid membrane (Horton *et al.*, 1991; Kim *et al.*, 2020). Consequently, examining the expression patterns of LHCSR1 and LHCSR3 homologs (potential candidates being Od17g02200, Od19g00040, and Od05g01870) and the STN7 homolog (Od09g02140), which is responsible for state transitions, could provide crucial insights to understand photosynthetic acclimation in *Ostreococcus*. Moreover, the decomposition of the up-regulated NPQ into specific quenching mechanisms (Pinnola and Bassi 2018) such as energy-dependent quenching (qE), zeaxanthin-dependent quenching (qZ), state transition (qT), and photoinhibition quenching (qI) will deepen our understanding of photosynthetic acclimation. Furthermore, biochemical and structural studies on the formation of LHC aggregates and PSII arrays, which are known to modulate their light-harvesting properties (Horton *et al.*, 1991; Kim *et al.*, 2020), may reveal underlying mechanisms.

In order to deepen our understanding of the genetic diversity and physiological responses in *Ostreococcus*, it is important to undertake extensive comparative studies. Such studies should include a variety of ecotypes and environmental conditions, as this will offer valuable insights for future research. In addition, the development of genetic engineering techniques for *Ostreococcus*, as demonstrated by Sanchez *et al.* (2019), is crucial for investigating phenotypic variation resulting from the absence of specific genes in order to elucidate the role of these genes *in vivo*. This research will contribute not only to our understanding of photosynthetic acclimation and evolutionary strategies but also to strategies to promote sustainable conservation and management of marine ecosystems.

## References

[CIT0001] Cardol P , Bailleul B, Rappaport F, et al. 2008. An original adaptation of photosynthesis in the marine green alga *Ostreococcus*. Proceedings of the National Academy of Sciences, USA 105, 7881–7886. 10.1073/pnas.0802762105 PMC2409423 18511560

[CIT0002] Courties C , Perasso R, Chrétiennot-Dinet M-J, Gouy M, Guillou L, Troussellier M. 1998. Phylogenetic analysis and genome size of *Ostreococcus tauri* (Chlorophyta, Prasinophyceae). Journal of Phycology 34, 844–849. 10.1046/j.1529-8817.1998.340844.x

[CIT0003] Courties C , Vaquer A, Troussellier M, Lautier J, Chrétiennot-Dinet M-J, Neveux J, Machado C, Claustre H. 1994. Smallest eukaryotic organism. Nature 370, 255–255. 10.1038/370255a0

[CIT0004] Derelle E , Ferraz C, Rombauts S, et al. 2006. Genome analysis of the smallest free-living eukaryote *Ostreococcus tauri* unveils many unique features. Proceedings of the National Academy of Sciences, USA 103, 11647–11652. 10.1073/pnas.0604795103 PMC1544224 16868079

[CIT0005] Hey D , Grimm B. 2018. ONE-HELIX PROTEIN2 (OHP2) is required for the stability of OHP1 and assembly factor HCF244 and is functionally linked to PSII biogenesis. Plant Physiology 177, 1453–1472. 10.1104/pp.18.00540 29930106 PMC6084673

[CIT0006] Horton P , Ruban AV, Rees D, Pascal AA, Noctor G, Young AJ. 1991. Control of the light-harvesting function of chloroplast membranes by aggregation of the LHCII chlorophyll protein complex. FEBS Letters 292, 1–4. 10.1016/0014-5793(91)80819-o 1959588

[CIT0007] Kim E , Watanabe A, Duffy CDP, Ruban AV, Minagawa J. 2020. Multimeric and monomeric photosystem II supercomplexes represent structural adaptations to low- and high-light conditions. Journal of Biological Chemistry 295, 14537–14545. 10.1074/jbc.RA120.014198 32561642 PMC7586214

[CIT0008] Krishnan-Schmieden M , Konold PE, Kennis JTM, Pandit A. 2021. The molecular pH-response mechanism of the plant light-stress sensor PsbS. Nature Communications 12, 2291. 10.1038/s41467-021-22530-4 PMC8052336 33863895

[CIT0009] Leliaert F , Smith DR, Moreau H, Herron MD, Verbruggen H, Delwiche CF, De Clerck O. 2012. Phylogeny and molecular evolution of the green algae. Critical Reviews in Plant Sciences 31, 1–46. 10.1080/07352689.2011.615705

[CIT0010] Lewis LA , McCourt RM. 2004. Green algae and the origin of land plants. American Journal of Botany 91, 1535–1556. 10.3732/ajb.91.10.1535 21652308

[CIT0011] Minagawa J. 2011. State transitions—the molecular remodeling of photosynthetic supercomplexes that controls energy flow in the chloroplast. Biochimica et Biophysica Acta 1807, 897–905. 10.1016/j.bbabio.2010.11.005 21108925

[CIT0012] Niyogi KK , Grossman AR, Björkman O. 1998. Arabidopsis mutants define a central role for the xanthophyll cycle in the regulation of photosynthetic energy conversion. The Plant Cell 10, 1121–1134. 10.1105/tpc.10.7.1121 9668132 PMC144052

[CIT0013] Niyogi KK , Li XP, Rosenberg V, Jung HS. 2005. Is PsbS the site of non-photochemical quenching in photosynthesis? Journal of Experimental Botany 56, 375–382. 10.1093/jxb/eri056 15611143

[CIT0014] Palenik B , Grimwood J, Aerts A, et al. 2007. The tiny eukaryote *Ostreococcus* provides genomic insights into the paradox of plankton speciation. Proceedings of the National Academy of Sciences, USA 104, 7705–7710. 10.1073/pnas.0611046104 PMC1863510 17460045

[CIT0015] Pinnola A. 2019. The rise and fall of light-harvesting complex stress-related proteins as photoprotection agents during evolution. Journal of Experimental Botany 70, 5527–5535. 10.1093/jxb/erz317 31424076

[CIT0016] Pinnola A , Bassi R. 2018. Molecular mechanisms involved in plant photoprotection. Biochemical Society Transactions 46, 467–482. 10.1042/BST20170307 29666217

[CIT0017] Sanchez F , Geffroy S, Norest M, Yau S, Moreau H, Grimsley N. 2019. Simplified transformation of *Ostreococcus tauri* using polyethylene glycol. Genes 10, 399. 10.3390/genes10050399 31130696 PMC6562926

[CIT0018] Sands E , Davies S, Puxty RJ, Verge V, Bouget F-Y, Scanlan DJ, Carre IA. 2023. Genetic and physiological responses to light quality in a deep ocean ecotype of Ostreococcus, an ecologically important photosynthetic picoeukaryote. Journal of Experimental Botany 74, 6773–6789. 10.1093/jxb/erad347 PMC10662239 37658791

[CIT0019] Six C , Sherrard R, Lionard M, Roy S, Campbell DA. 2009. Photosystem II and pigment dynamics among ecotypes of the green alga *Ostreococcus*. Plant Physiology 151, 379–390. 10.1104/pp.109.140566 19587099 PMC2735990

[CIT0020] Theis J , Schroda M. 2016. Revisiting the photosystem II repair cycle. Plant Signaling and Behavior 11, e1218587. 10.1080/15592324.2016.1218587 27494214 PMC5058467

